# Effectiveness of individualized 3D titanium-printed Orthognathic osteotomy guides and custom plates

**DOI:** 10.1186/s12903-023-03000-3

**Published:** 2023-05-03

**Authors:** Se-hyang Kim, Sung-Min Lee, Jung-Hyun Park, Sook Yang, Jin-Woo Kim

**Affiliations:** 1 Graduate School of Clinical Dentistry, Ewha Womans University, Seoul, South Korea; 2 Department of Oral and Maxillofacial Surgery, School of Medicine, Ewha Womans University, Anyangcheon-Ro 1071, Yangcheon-Gu, Seoul, 158-710 Korea; 3 Cusmedi Co., Ltd., Gyeonggi-Do, Suwon-Si, 400815 South Korea

**Keywords:** Orthognathic surgery, Virtual surgery, Computer-aided surgical simulation, Patient customized implant, Titanium printing

## Abstract

**Background:**

Computer-aided design/manufacturing (CAD/CAM) technology was developed to improve surgical accuracy and minimize errors in surgical planning and orthognathic surgery. However, its accurate implementation during surgery remains a challenge. Hence, we compared the accuracy and stability of conventional orthognathic surgery and the novel modalities, such as virtual simulation and three-dimensional (3D) titanium-printed customized surgical osteotomy guides and plates.

**Methods:**

This prospective study included 12 patients who were willing to undergo orthognathic surgery. The study group consisted of patients who underwent orthognathic two-jaw surgery using 3D-printed patient-specific plates processed by selective laser melting and an osteotomy guide; orthognathic surgery was also performed by the surgeon directly bending the ready-made plate in the control group. Based on the preoperative computed tomography images and intraoral 3D scan data, a 3D virtual surgery plan was implemented in the virtual simulation module, and the surgical guide and bone fixation plate were fabricated. The accuracy and stability were evaluated by comparing the results of the preoperative virtual simulation (T0) to those at 7 days (T1) and 6 months (T2) post-surgery.

**Result:**

The accuracy (ΔT1‒T0) and stability (ΔT2‒T1) measurements, using 11 anatomical references, both demonstrated more accurate results in the study group. The mean difference of accuracy for the study group (0.485 ± 0.280 mm) was significantly lower than in the control group (1.213 ± 0.716 mm) (*P* < 0.01). The mean operation time (6.83 ± 0.72 h) in the control group was longer than in the study group (5.76 ± 0.43 h) (*P* < 0.05).

**Conclusion:**

This prospective clinical study demonstrated the accuracy, stability, and effectiveness of using virtual preoperative simulation and patient-customized osteotomy guides and plates for orthognathic surgery.

## Introduction

Orthognathic surgery is used to improve the oral and facial function and esthetics of patients by correcting the imbalances associated with craniofacial structures and skeletal malocclusion. Restoration of normal jaw function, optimal facial esthetics, and long-term stability are the goals of orthognathic surgery [1, 2]. The key factors that determine the success of orthognathic surgery are the optimal diagnosis, treatment planning, and accurate surgical delivery of the preoperative simulation to the operating room. However, while maxillofacial surgeons strive to provide the ideal treatment for patients with functional and esthetic discomfort, the accurate delivery of a preoperative surgical plan to actual surgery is challenging.

During orthognathic surgery, the maxilla is repositioned to the preplanned location through intentional LeFort I osteotomy before performing the mandibular osteotomy, which has been determined by the prefabricated surgical wafers and internal or external references of the skull for the horizontal and vertical osteotomy. However, considerable errors are known to occur during this process, and even a few millimeters of error can cause serious surgical failures in orthognathic surgery [3]. Since accurate maxillary positioning during surgery is a crucial aspect in reproducing the surgical plan, there is an urgent need for improving intraoperative accuracy.

Recently, the development of computer-aided design/manufacturing (CAD/CAM) and three-dimensional (3D) printing technology has attracted much attention as a modality for improving intraoperative accuracy [4]. CAD/CAM technology has enabled preoperative virtual simulation according to the treatment plan [5], while 3D printing technology plays a role in the application of virtual preoperative simulation to the surgical field. In addition, 3D-metal printing using selective laser sintering allows the fabrication of individualized bone fixation plates and bone reconstruction materials [6, 7]. Although several studies have described computer-assisted virtual planning for orthognathic surgeries [8-11], the accurate application of the prefabricated device to the determined location during surgery remains a challenge, as does the lack of any evaluation of the effectiveness and accuracy of the 3D-printed plates and osteotomy guides to ensure they are correct for surgical implementation.

Therefore, the purpose of this study was to compare the accuracy, stability, and effectiveness of conventional orthognathic surgery and the virtual simulation combined with 3D-printed patient-customized surgical guides and plates.

## Methods

### Study design

This prospective clinical study was performed from 2019 to 2021 in the departments of Orthodontics, and Oral and Maxillofacial Surgery at the University Hospital, Seoul, South Korea. The patients were selected according to the following inclusion criteria: (1) patients who were scheduled to undergo orthognathic surgery between 2019 and 2021; (2) patients who had a Class III malocclusion and had undergone Le Fort 1 maxillary osteotomy, or bilateral sagittal split ramus osteotomy, with or without genioplasty. The exclusion criteria were as follows: (1) patients with cleft palate or other craniofacial anomalies; (2) patients who were unwilling to participate in this study. The study group consisted of patients who underwent orthognathic surgery using an osteotomy guide and customized titanium plates processed by selective laser melting (SLM). For the control group, readymade titanium plates were manually contoured to fit the jaw anatomy. Computer-aided surgical simulation and the fabrication of intermaxillary wafers were performed before orthognathic surgery for both groups. All medical practices conformed to the Declaration of Helsinki. The study protocol was approved by the hospital’s Institutional Review Board (IRB No. 2019–06-014). All patient data were anonymized and deidentified prior to analysis.

### 3D image acquisition

DICOM data on the cone-beam computed tomography (CBCT) were extracted into the STL format and merged with the intraoral scanning STL data. The CBCT dataset, obtained 2 weeks before surgery, was surface-rendered in the 3D model (STL format) of the bone. CBCT was taken using CS 9600 (Carestream, Inc., Ilkley, UK), with a matrix size of 512 × 512, a voxel size of 300 µm, a layer thickness of 0.5 mm, and a field of view (FOV) of 16 × 17 cm. Intraoral scanning with Trios3 (3 shape, Copenhagen, Denmark) started with the most distal tooth in the third quadrant and continued to the anterior teeth. Next, the fourth quadrant was scanned, again beginning with the most distal tooth. Scanning of the maxilla started with the most distal tooth in the second quadrant and ended at the central incisor. The first quadrant was recorded, starting with the most distal tooth. The camera was positioned at 45 degrees (or as close as possible to the axis of the tooth) to the buccal and lingual scans. The scanning device worked by means of confocal microscopy, with a fast scanning time; the light source provided an illumination pattern to cause a light oscillation on the object. The DICOM data of the patient were converted into a 3D model with an STL (Standard Tessellation Language) format using the Aview Modeler software (Aview Modeler, Corelinesoft. LTD, Seoul, South Korea). The 3D model was extracted by adjusting the range of the threshold limits. The image segmentation was offered by the Aview Modeler software, including automatic thresholding and minor manual corrections. The 3D models of the bones were generated and exported in STL format files.

Then, the intraoral 3D scan data were digitized into the Surface Tessellation Language format using a scanner Trios3 (3 shape, Copenhagen, Denmark). The CBCT images were transformed into a DICOM format, and three-dimensionally reconstructed. Subsequently, the DICOM and STL files were imported into a planning software program. The patient’s CBCT scan and the scanned image of the patient’s dental cast were registered. Semi-automatic merging started with registering a tooth image obtained from an intraoral scanner to a relatively accurate CBCT image of the tooth. The images were merged via manual registration, by selecting three anatomical landmarks from the dentition. The contour of the dental cast image placed on the CBCT image was examined, and fine adjustments were made, if necessary. Thus, the final virtual hybrid skull-dentition 3D image (virtual face) was obtained. To improve the accuracy of the fusion of the 3D skull STL and intraoral scanning data, the 3D skull STL data were automatically aligned with the bone mandibular condyles, and the upper incisors were used as reference points. After alignment, the intraoral scan data were matched to the correct position with the STL model for the skull using the “registration tool” of software “meshlab” (open-access software, Italy).

For the superimposition and merger of DICOM + STL (including software info), a 3D Slicer (extension slicer RT, ver.4.11, open source) was used to create the 3D bone model file (STL format), while Meshmixer (ver.3.5, Autodesk) was used to edit the 3D slicer-created surface model.

Using Ondemand® CAD/CAM software (Cybermed Co., Seoul, Korea) and Doctor Check software® (Cusmedi Co., Seoul, Korea), the virtual miniplates and osteotomy guides were designed with respect to the patient’s bone contour and individual surgical location. The proper position was determined preoperatively on CBCT, after considering the bone density for fixational support and avoiding any adjacent tooth injury.

**Fig. 1:**
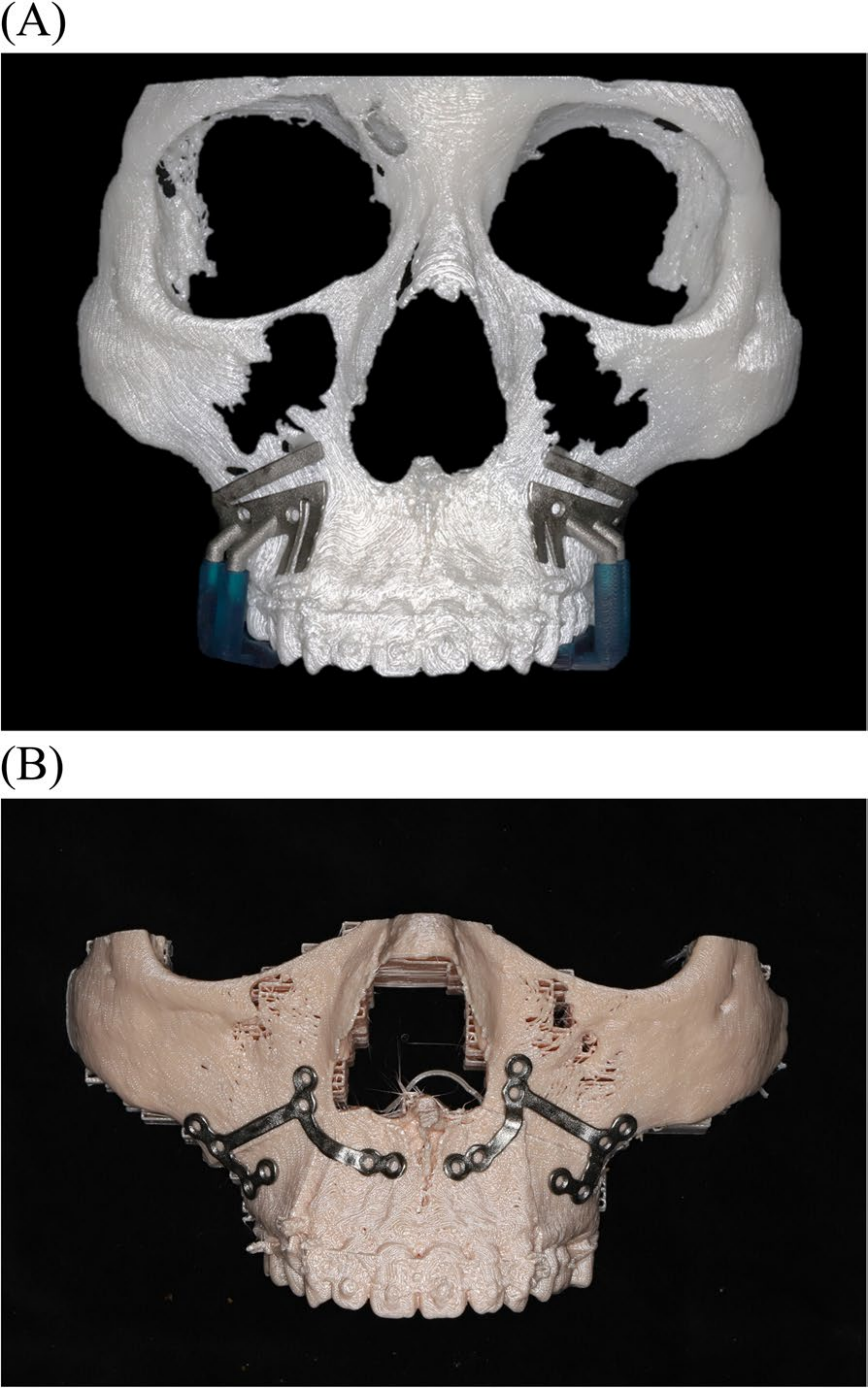
3D printed patient-specific maxillary osteotomy guide (**A**) and plates (**B**). A customized maxillary osteotomy guide combined with a blue resin 3D printed tooth guide jig for delivery on the exact simulated position

The surgeon conducted 3D rendering, preoperative virtual simulation, and designed the individualized plates and osteotomy guides. These customized plates and osteotomy guides were exported in an STL format from the CAD software program (Magics, Materialise, Leuven, Belgium). The 3D titanium printing was performed using an SLM 3D printer (Metalsys150, Winforsys Co., Seoul, Korea; laser power 120–200 W; Z-axis Travel accuracy is ± 3 μm, the beam spot is ~ 70–150 μm) laminated by irradiating the powder bed with a laser (Ti6Al4V ELI, medical grade in accordance with ASTM F136& F3001, AP&C, Quebec, QC, Canada) to instantaneously melt a local area and induce powder interlayer welding. The SLM 3D printer completely melted and bonded the metal powder and compressed it perfectly with excellent strength and precision. The process of 3D printing consisted of three steps: modeling, printing, and postprocessing. Postprocessing of the manufactured guides and plates involved grinding, washing, and drying the final medical equipment, after which, it was transported to a surgeon. During postprocessing, polishing of Ra < 15 um was conducted. Based on ISO 17665–1, 2, the autoclave process was also performed as a final step. A rapid prototype model was fabricated to evaluate adaptability before surgery (Fig. 1).

### Surgical procedure

**Fig. 2:**
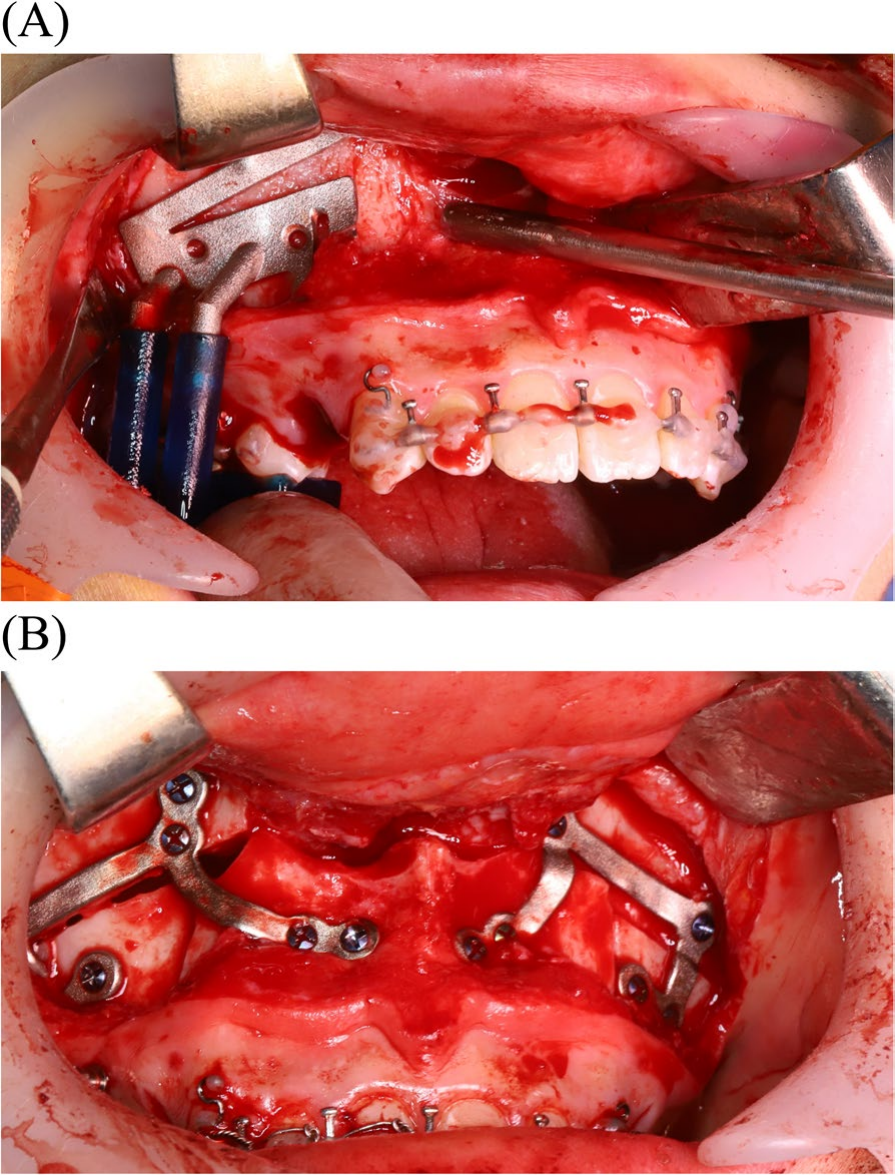
**A** 3D printed patient-specific guide applied to the maxilla. LeFort I and segmental maxillary osteotomy were planned. **B** 3D printed patient-specific fixation plates in place

To minimize the occurrence of potential errors arising from CBCT acquisition, virtual simulation, and 3D printing during the fabrication and actual placement of the osteotomy guide and miniplates, the authors fabricated surgical guides based on the adjacent teeth to maximize the adaptation accuracy. The use of CBCT imaging alone is insufficient to accurately visualize the dentition due to inaccurate rendering of the teeth and streak artifacts caused by dental restorations or metal orthodontic appliances. Therefore, in order to manufacture a surgical wafer with a surgical occlusion plan (CAD/CAM) method during virtual surgery, a process of matching with the intraoral scan data was required to compensate for the low resolution of the CBCT data [12, 13]. The osteotomy guides were designed to be fixed with additional screw holes to minimize their mobility during sawing (Fig. 2A). After the adaptation of the osteotomy guides, based on the adjacent teeth, the osteotomy guide with monocortical screws (Osteonic orthognathic surgery system, Seoul, South Korea), and intentional Lefort I osteotomy were performed. Osteotomy was conducted using a 1.05 × 70 mm reciprocating surgical saw (Stryker, Portage, MI, United States).

Three-dimensional intermaxillary, craniomaxillofacial relationships and soft tissue profiles used to achieve the ideal facial esthetics and occlusion were considered to establish the surgical treatment objectives (STOs). The STOs were determined through an agreement between the orthodontists and oral surgeons, then, the plan was transferred to the virtual simulation of the jaws. Two oral surgeons and one orthodontist participated in this process for the patients involved in this study. After osteotomy, the jaw segments were reduced using a surgical wafer, which possessed the STO information. The surgical wafers were prefabricated using 3D printing based on a virtual simulation. The surgical wafer for the orthognathic surgery consisted of an intaglio part for the teeth, thus, the jaw, which is connected to the teeth, was seated on the surgical wafer in order to be correctly reduced. After the maxillary repositioning at the planned location, an intermediate surgical wafer was inserted, and the individualized plates were placed in a preoperatively simulated position based on the vertical buttress of the maxilla. Then, the individualized plates were adapted and fixed with monocortical screws (Fig. 2B). A unilateral sagittal split ramal osteotomy was performed in the same way as for the mandibular surgery and the maxillary surgery. The mandible ramus was cut obliquely according to the adjacent tooth reference guide, divided into two plates, and moved to the most ideal position, while the individualized plates were adapted and fixed with monocortical screws.

### Outcome evaluation

We evaluated the treatment modalities for accuracy, stability, and effectiveness. Accuracy and stability were evaluated by comparing the preoperative virtual simulation to the results taken at 7 days and 6 months postoperation. The CBCT images taken before surgery (T0) and 7 days postoperatively (T1), and 6 months postoperatively (T2) were converted into a STL format. Eight anatomical landmarks, which were used to measure the linear differences, were both central points between the cusp ends of the maxillary central incisors, both cusp ends of the maxillary canines, both mesial cusps of the maxilla first molars, and both midpoints of the maxilla bone nasal notch. In addition, three mandibular anatomical landmarks—both mental foramen and point B—were used.

**Fig. 3:**
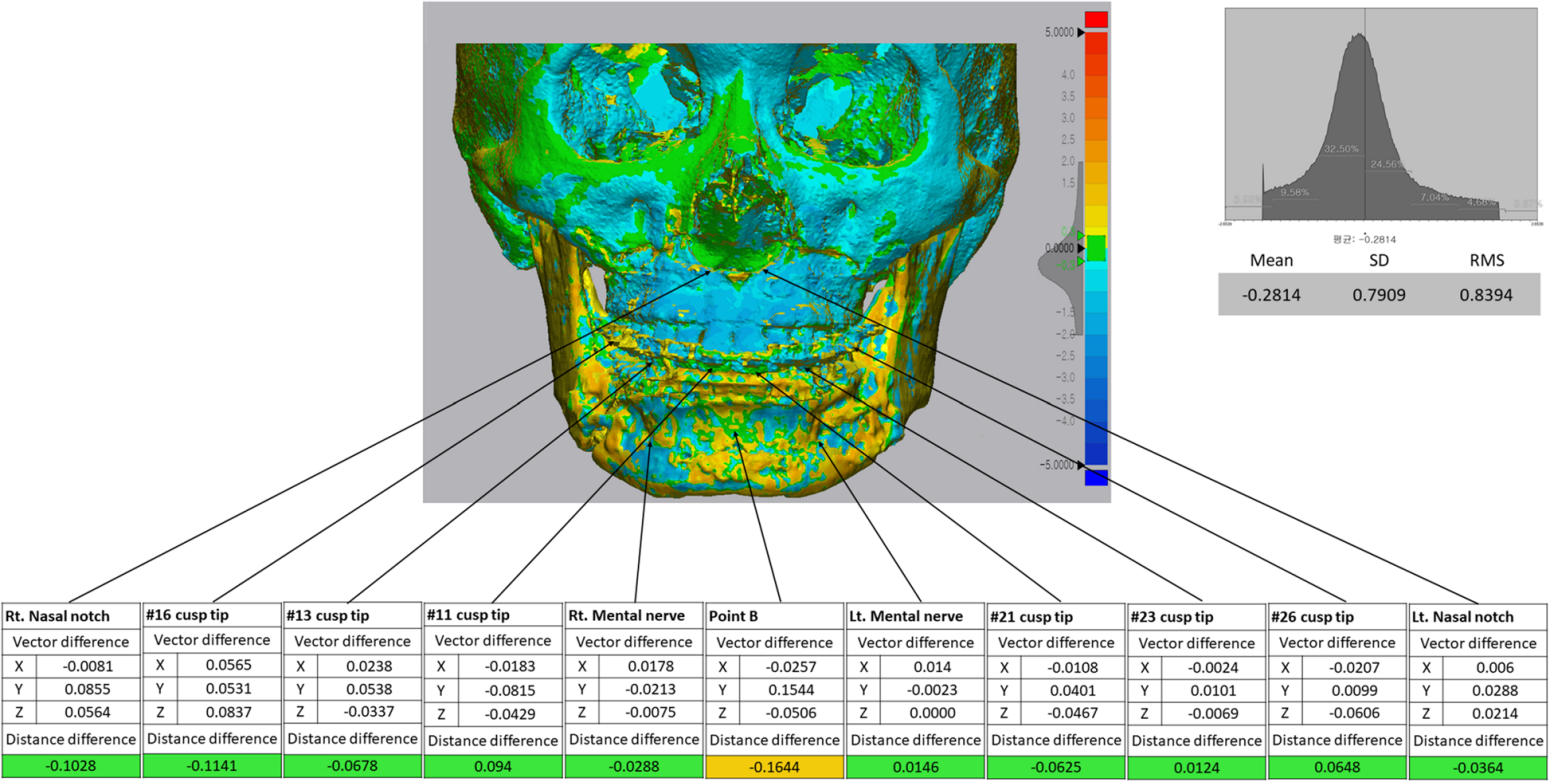
Superimposition of preoperative virtual simulation and actual surgery. Linear differences were measured. Color grade visualized the difference ranging from -5 mm (blue) to + 5 mm (red)

Accuracy was evaluated by superimposing the preoperative simulation (T0) and the CT data on the 7^th^ day after the operation (T1). To match the preoperative simulation and the 7-day postoperative CBCT, registration was performed by setting the surgically unaffected area as the reference point. The preset fiducials used for registration were the frontozygomatic suture, infraorbital foramen, and nasal notch midpoint, using Geomagic Verify® software (Freeform Plus, 3D Systems, North Carolina, USA). Further, the linear differences between the planned and actual movements were evaluated. The distance of each landmark set in the simulated predictive image (T0) and the CBCT image at 7 days post-surgery (T1) was measured based on the X, Y, and Z axes (Fig. 3). The automatic analysis function that calculates and displays the deviation between the reference and measurement model of the 3D measurement software (Geomagic) was used, and the coordinate system of the X, Y, and Z axes was automatically established. A preplanned preoperative simulation STL file was used as the reference data. The superimposed data were arranged in a three-dimensional space, and the coordinate values of the X, Y, and Z axes were automatically set and calculated. To measure the linear differences, we measured the root-mean-square deviation, mean, standard deviation, and 95% confidence intervals along the X, Y, and Z axes. Based on previous studies, it was assumed that the mean of the linear discrepancy between the hypothetically predicted and the actual postoperative outcome should not exceed the clinically acceptable value of 2 mm [14-16]. The deviation values among the data are displayed through color grades, which range from -5 mm (blue) to + 5 mm (red). We analyzed the differences in dentition and between the aligned and surgically moved bone surfaces, focusing on bone structures without direct surgical movements, such as the orbital region, infraorbital foramen, zygomatic bone, and nasal bone.

Surgical stability was measured by superimposing CT data at 7 days (T1) and 6 months (T2) after surgery to evaluate the surgical relapse after orthognathic surgery. Using Geomagic Verify® software (Freeform Plus, 3D Systems, North Carolina, USA), the linear differences between the immediate postoperative and 6 months after were examined. We investigated any complications, such as postoperative infection, soft tissue problems, reoperation, and adverse reactions to medical devices.

For effectiveness, the surgeons were surveyed to evaluate the effectiveness of the individualized guides and plates compared to conventional surgery. The questionnaire consisted of a total of 8 items, on a Likert 7-point scale ranging from 1 point for 'not at all' to 7 points for 'strongly agree.' The questionnaire items included the ease and fit of the patient-customized guides and metal plates, the degree of convenience for the operator compared to the conventional metal plate-applied surgery, the change in operation time, and overall satisfaction.

The data were analyzed using Statistical Package for Social Sciences (SPSS, version 26.0, IBM Co., Armonk, NY, USA). Differences between preoperative (T0) and the results at 7 days (T1) and 6 months (T2) post-surgery were evaluated using a paired t-test. A value of *P* < 0.05 was considered significant.

## Results

A total of 12 patients were included in the study (Table 1). The study group included 6 patients (3 females and 3 males; mean age: 20 years) and the control group included 6 patients (4 females and 2 males; mean age: 21 years). All patients presented skeletal and dental Class III malocclusion and underwent orthognathic surgery: Le Fort 1 osteotomy, and bilateral sagittal split osteotomy.

**Table 1:** Characteristics of participated patients and surgery

Patient No	Age	Sex	Skeletal Angle Classification	Amount of preoperative crossbite	Maxillary surgery	Mandibular surgery
1	23	F	Class 3	- 3 mm	Impaction 4 mm, advance 2 mm	Set-back 5 mm
2	15	F	Class 3	- 11 mm	ANS down 1.5 mm PNS imp 3.5 mm, adv 3 mm	Set-back 12 mm
3	17	F	Class 3	- 8 mm	Impaction 3 mm, advance 6.6 mm	Set-back 10.5 mm
4	19	M	Class 3	- 8 mm	Impaction 6 mm, advance 2 mm, yawing correction	Set-back 11 mm
5	21	M	Class 3	- 3 mm	Impaction 3 mm, advance 2 mm	Set-back 5 mm
6	27	M	Class 3	- 5 mm	Impaction 3 mm, advance 2 mm	Set-back 6 mm
7	19	F	Class 3	- 1.5 mm	Impaction 4 mm, advance 2 mm, canting correction	Set-back 3 mm, clockwise rotation
8	20	F	Class 3	- 3.5 mm	Impaction 3 mm, advance 5 mm	Set-back 6 mm
9	23	F	Class 3	- 3 mm	ANS 1 mm, PNS impaction 3 mm	Set-back 6 mm
10	21	M	Class 3	- 4 mm	ANS down 1 mm, PNS imp 2 mm, canting correction	Set-back 6.5 mm
11	22	F	Class 3	- 6 mm	ANS PNS imp 4 mm, adv 2 mm, translation 2.5 mm	Set-back 9.5 mm
12	21	M	Class 3	- 2 mm	ANS imp 4 mm, setback 2 mm, yawing correction	Set-back 4 mm

Accuracy was evaluated by comparing the preoperative simulation and the CT data on the 7^th^ day after the operation (ΔT1‒T0). The study group showed a higher accuracy in the results for the mean difference in distance at all anatomical reference points between T0 and T1 (0.485 ± 0.280 mm), compared to the control group (1.213 ± 0.716 mm) (*P* < 0.01; Table 2). Measuring the difference between T1 and T2, in terms of stability, revealed a mean difference in the distance of 0.529 ± 0.248 mm in the study group and 1.124 ± 0.193 mm in the control group, which was similar to the above results (*P* < 0.01; Table 3).

**Table 2:** Accuracy measurements of difference between preoperative virtual simulation and actual surgery (ΔT1 (T0‒T1)) of study and control groups (*n* = 12)

	Study group ΔT1 (T0‒T1)		Control group ΔT1 (T0‒T1)		P
	Mean	SD	Mean	SD	
Rt. Nasal notch	0.371	0.299	1.008	1.050	n.s
Lt. Nasal notch	0.476	0.480	0.544	0.573	n.s
#16 cusp tip	0.111	0.139	1.204	0.810	0.03
#13 cusp tip	0.607	0.704	1.553	0.500	< 0.01
#11 cusp tip	0.441	0.674	1.243	0.571	0.02
#21 cusp tip	0.359	0.530	1.371	0.632	< 0.01
#23 cusp tip	1.046	0.776	1.004	0.389	< 0.01
#26 cusp tip	0.346	0.454	0.825	0.735	n.s
Point B	0.692	0.505	1.735	0.851	< 0.01
Rt. mental nerve	0.478	0.298	0.851	0.586	n.s
Lt. mental nerve	0.410	0.305	2.005	0.981	< 0.01
Total	0.485	0.280	1.213	0.716	< 0.01

*Abbreviations*: *n.s* not significant, *Lt* left, *Rt * right, *SD* standard deviation

**Table 3:** Stability measurements of differences between superimposition CT data at 7 days (T1) and 6 months (T2) after surgery of study and control groups (*n* = 12)

	Study group ΔT2 (T1‒T2)		Control group ΔT2 (T1‒T2)		P
	Mean	SD	Mean	SD	
Rt. Nasal notch	0.322	0.368	1.290	0.928	0.02
Lt. Nasal notch	0.444	0.378	0.697	0.160	n.s
#16 cusp tip	0.293	0.125	0.499	0.403	n.s
#13 cusp tip	0.411	0.568	0.856	0.376	n.s
#11 cusp tip	0.251	0.217	1.447	0.712	< 0.01
#21 cusp tip	0.588	0.733	1.406	0.628	n.s
#23 cusp tip	1.015	0.497	0.914	0.559	n.s
#26 cusp tip	0.396	0.457	0.625	0.310	n.s
Point B	0.729	0.514	1.521	0.874	n.s
Rt. mental nerve	0.643	0.556	1.297	1.092	n.s
Lt. mental nerve	0.729	0.440	1.801	0.815	0.04
Total	0.529	0.248	1.124	0.193	< 0.01

*Abbreviations*: *n.s* not significant, *Lt* left, *Rt * right, *SD* standard deviation

Significant complications, such as nerve injury, tooth loss, postoperative relapse or malocclusion, and infection were not observed during the follow-up period, except in one patient. In this patient, exposure of the right mandibular plate and surrounding inflammatory tissue was observed postoperatively at 8 weeks. The stability of the bone segment was acceptable without any evident bone resorption. Surgical debridement and changes to the conventional plate were conducted as the exposure persisted even after minimal debridement and 2 weeks of antibiotic therapy. However, during the 6-month follow-up, normal healing was observed.

The mean operation times (based on electronic medical records measuring the time from entering the operating room to leaving the room) were 6.83 ± 0.72 h and 5.76 ± 0.43 h for the control and study groups, respectively (*P* < 0.05). Interestingly, the time for selection and adaptation of the miniplates on the bone contour, the adjustment of the miniplates, and the confirmation of the location of the repositioned bone were minimized. The additional time and costs of the virtual device design and fabrication were not considered for the above-mentioned operation times.

## Discussion

This prospective clinical study investigated the accuracy, stability, and efficiency of virtual preoperative simulation combined with patient-customized osteotomy guides and plates compared to conventional orthognathic surgery. Virtual preoperative simulations facilitate precise diagnosis and treatment planning, while the integration of 3D metal printing technology enables accurate implementation of the treatment plan in the operation. To our knowledge, this is the first study comparing the accuracy and stability of orthognathic surgery between conventional orthognathic surgery and virtual simulation combined with 3D titanium-printed patient-customized surgical guides and plates.

As ANS, PNS, and point A are parts that can be removed during surgery, the nasal notch of the maxilla bone was used as an alternative reference point. Rotation of both jaws may occur in conventional orthognathic surgery when an intermaxillary wafer is used to position the maxilla based on the mandible, which can lead to postoperative variation [17]. Since the mandibular condyle translates and rotates within the temporomandibular joint fossa [18], repositioning the maxilla according to the mandible using an intermaxillary wafer frequently leads to errors. In addition, intermaxillary wafers can be inaccurate even at the model surgery stage. Accurately reproducing virtual surgery during actual surgery depends on optimal intermaxillary relations, occlusion, and face bow transfers, which record the relationship between the maxilla and the hinge axis of the mandible rotation. Ellis et al. reported an inaccuracy of nearly 7 degrees in the angle of the occlusal plane during face bow transfer [19]. Baily et al. found a mean difference of 5 degrees in the angle difference of the occlusal plane to the Frankfort plane in the Hanau articulator, which is 70% of the face bow transfer error [20]. Considering the inaccuracy in the recording processes, the use of a 3D printed plate and guide in the preoperative simulation can reduce the errors related to model surgery because an articulator is not used.

Rustemeyer et al. reported that 2D cephalometric analysis and 3D simulation are sufficient for accurate planning [21]. In orthognathic surgery with complex surgical options, such as yawing and canting correction, the conventional method with an articulator can make many errors, while the 3D virtual simulation enabled the establishment of accurate preoperative planning and the fabrication of wafers [22].

The customized printed plate has high plasticity and a patient-customized manufacturing process, which reduced the fatigue caused by plate bending [23].

The accuracy of 3D printed plates may vary when manufactured through 3D printing, depending on the initial resolution of the 3D image. It is possible to manufacture a refined (delicately trimmed) plate according to the patient's bone surface, which will present a compatibility with the bone. Customized plates are highly rigid, which enables the correct repositioning of bone segments and the ability to withstand functional loads.

The operation time is shortened by using a 3D printer to manufacture a customized metal plate and a guide device for osteotomy, movement, and fixation, enabling the jawbone movement as planned. We found some limitations in this modality after performing several surgeries. There was a risk of infection with the customized plates: One patient who underwent orthognathic surgery with the customized plates presented some inflammation in the right mandible. We speculate that inflammation may occur in oral tissues depending on the roughness of the plate surface. Hence, the plate surface roughness must be standardized. Problems may arise in the 3D simulation planning or in communication between the operator and the engineer, which may lead to errors in custom plate settling. 

Following the analysis of the variables, including the differences in distance, different results can be obtained depending on the analyst, even if the location of the same landmark is specified. In comparative analysis focusing on teeth, including the landmarks on the maxilla–for example, cusps and apex, errors may occur in the analysis values due to orthodontic tooth movement after OP (T2).

This study has a limitation, whereby the patients and surgeons did not process the data ‘blind’, which can induce possible bias. Further studies with a blindly controlled trial setting and increased sample size are needed.

In conclusion, this prospective clinical study demonstrated the accuracy, stability, and efficiency of virtual preoperative simulation and patient-customized osteotomy guides and plates for orthognathic surgery. Virtual preoperative simulations ensured precise diagnosis and treatment planning, while the integration of 3D metal printing technology enabled accurate delivery of treatment plans to the actual operation. The use of individualized 3D titanium-printed tooth-referenced orthognathic osteotomy guides and plates might help improve surgical accuracy and prognosis and decrease operation times.

## Data Availability

The datasets generated during and/or analyzed during the current study are available from the corresponding author upon reasonable request.
